# Sotagliflozin corrects myocardial phenotype, mitochondrial gene expression and oxidative stress in mice with hypertrophic cardiomyopathy^☆^

**DOI:** 10.1016/j.jmccpl.2026.100873

**Published:** 2026-10-12

**Authors:** Fuzhong Qin, David R. Pimentel, Tomas Baka, Dominique Croteau, Richard A. Cohen, Ivan Luptak, Wilson S. Colucci

**Affiliations:** Cardiovascular Medicine Section, Myocardial Biology Unit, and the Clinical and Translational Institute, Boston University School of Medicine, Boston, MA, United States of America

**Keywords:** SGLT2 inhibitor, Heart failure, Myocardial hypertrophy, Myocardial fibrosis, SarcoEndoplasmic Calcium ATPase, Diastolic dysfunction

## Abstract

**Background:**

Hypertrophic cardiomyopathy (HCM) is associated with mitochondrial dysfunction and oxidative stress.

**Objective:**

Our goal was to determine the effects of the dual Sodium-Glucose-Linked-Transporter (SGLT) 1 and 2 inhibitor sotagliflozin (SOTA) on myocardial remodeling, diastolic function, mitochondrial gene expression and oxidative stress in mice with HCM due to the tropomyosin E180G mutation (E180G).

**Methods:**

E180G and non-carrier (NC) mice 7–8 weeks old were randomized to SOTA formulated in the chow (30 mg/kg chow) or control diet (CD) for 8 weeks. Cardiac structure and function were assessed by echocardiography at baseline, and after 4 and 8 weeks of treatment. At 8 weeks mice were sacrificed to assess myocardial histology, RNA expression and oxidative stress.

**Results:**

At baseline E180G mice had myocardial hypertrophy and diastolic dysfunction that worsened over the next 8 weeks. In E180G mice, treatment with SOTA for 8 weeks prevented further LV hypertrophy, decreased interstitial fibrosis, normalized diastolic function and decreased atrial size. In E180G hearts mitochondrial gene sets were downregulated and oxidative stress was increased. SOTA treatment upregulated mitochondrial gene sets and prevented the increase in oxidative stress. Upregulated fibrosis gene sets in E180G hearts were not affected by SOTA.

**Conclusions:**

In a mouse model of HCM, SOTA exerts beneficial effects on myocardial structure and diastolic function which are associated with restoration of down-regulated mitochondrial gene programs and correction of oxidative stress. SOTA may be of clinical value in the treatment of patients with HCM.

## Highlights

• Hypertrophic cardiomyopathy (HCM) is associated with mitochondrial dysfunction, impaired myocardial energetics and excessive release of reactive oxygen species (ROS).
• SGLT2 inhibitors may up-regulate mitochondrial gene programs, improve myocardial energetics and decrease ROS.
• Mice with a E180G mutation in tropomyosin have an HCM phenotype with myocyte hypertrophy, interstitial fibrosis and diastolic dysfunction that is associated with downregulation of mitochondrial gene programs and increased ROS.
• Treatment with the SGLT1/2 inhibitor sotagliflozin (SOTA) for 8 weeks prevented further LV hypertrophy, decreased interstitial fibrosis, normalized diastolic function, upregulated mitochondrial gene programs and decreased markers of oxidative stress.
• These findings suggest that SOTA may be of clinical value in the treatment of patients with HCM, and further, support a central role for mitochondrial dysfunction in the pathophysiology of HCM.

## Graphical abstract

**Figure:**
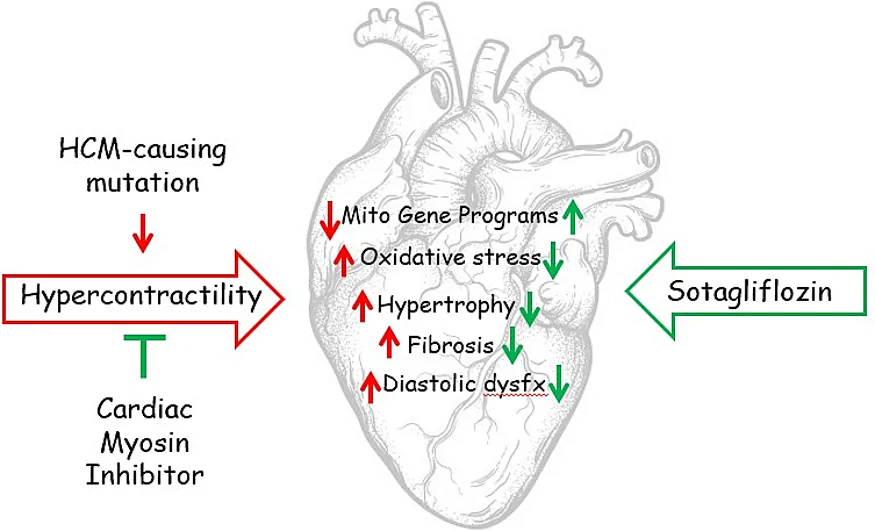

## 1 Introduction

The hypertrophic cardiomyopathy (HCM) phenotype is characterized by myocardial hypertrophy, fibrosis, impaired relaxation and mitochondrial dysfunction with impaired oxidative metabolism and oxidative stress. We recently found that the SGLT2 inhibitor empagliflozin (EMPA) improved the myocardial phenotype in mice with HCM caused by a mutation of myosin at the 403 codon (R403Q) with substitution of glutamine for arginine [1]. While these findings have potentially important implications for the use of SGLT2 inhibitors in patients with HCM, it is not known whether these initial observations can be generalized to other mouse models of HCM or other SGLT2 inhibitors. Our first goal was to determine whether the beneficial effects we observed with EMPA in the myosin R403Q mouse can be replicated with a different mouse model and SGLT2 inhibitor. Accordingly, we evaluated the effects of sotagliflozin (SOTA) on the cardiac phenotype of mice with HCM caused by a mutation of tropomyosin at the 180 codon (E180G) resulting in substitution of glycine for glutamic acid [2], [3], [4]. The E180G mouse replicates the classic features of HCM with myocardial hypertrophy, impaired relaxation and fibrosis [5]. Here we show that SOTA, an SGLT inhibitor that differs from EMPA in SGLT1/2 selectivity [6] and lipophilicity [7] prevented or improved the central features of the HCM phenotype – myocardial hypertrophy, interstitial fibrosis and diastolic dysfunction. These findings thus suggest that the benefits of SGLT inhibition in HCM are not specific to the HCM model or gliflozin.

Our second goal was to determine whether the beneficial effects of SOTA are related to improvements in mitochondrial gene expression and oxidative stress. Studies in myocardium from patients and mouse models of HCM have shown mitochondrial dysfunction [8], [9] associated with down-regulation of gene pathways for mitochondrial structure and function [1], [10], [11]. Mitochondrial dysfunction may affect the HCM phenotype both by limiting the function of multiple ATP-dependent proteins and via the effects of excessive ROS that cause oxidative post-translational modifications (OPTM) of proteins involved in mediating key cellular processes including remodeling [12], calcium homeostasis [13], sarcomere function [14], [15] and energetics [16].

We previously found that an SGLT2 inhibitor, ertugliflozin (ERTU), upregulated myocardial gene expression for mitochondrial pathways for oxidative phosphorylation and fatty acid metabolism in mice with diastolic dysfunction due to diet-induced obesity [17] or systolic dysfunction due to cardiac-specific overexpression of Gαq [18], and in both cases was associated with correction of mitochondrial reactive oxygen species (ROS) release. In the current study with E180G mice, marked downregulation of myocardial gene programs for oxidative phosphorylation and fatty acid oxidation was associated with oxidative stress. SOTA treatment both upregulated mitochondrial gene expression and corrected oxidative stress.

## 2 Methods

### 2.1 Experimental animals and study design

Male and female mice with the tropomyosin E180G mutation and non-carrier (NC) mice [3], [4] were obtained from Jackson Laboratories (Bar Harbor, ME) at 5–6 weeks of age and randomized at 7–8 weeks of age to SOTA formulated in the chow (30 mg/kg chow) or control diet (CD) for 8 weeks. Body weight was measured weekly. Echocardiography was performed at baseline, 4 and 8 weeks of treatment, and all mice were sacrificed at 8 weeks of treatment for cardiac weights, histology and RNA sequence analysis. The protocol was approved by the Institutional Animal Care and Use Committee at Boston University School of Medicine.

### 2.2 Echocardiography

Two-dimensional, M-mode and Doppler echocardiography were used to measure cardiac function and dimensions using a VisualSonics Vevo 3100 high-resolution imaging system equipped with a 22- to 55-mHz MS550D transducer, as previously described based on the average of at least 5 consecutive cardiac cycles in each animal [17].

### 2.3 Histology and immunohistochemistry

A piece of LV from each mouse was fixed in 10% buffered formalin and then embedded in paraffin, sectioned, stained and analyzed, as we have described [19]. Myocyte cross-sectional area was measured using National Institutes of Health (NIH) ImageJ software as we have described in 60 myocytes per animal [19]. Fibrosis was assessed by staining sections with a Picrosirius Red Kit (Polysciences), as we have described, in four random fields in each of 4 sections from each animal [19]. 4-hydroxynonenal (HNE) immunohistochemical staining was performed using a mouse anti-HNE monoclonal antibody (Oxis Research), as we have described, in twenty images selected from 4 sections from each animal [19]. Sulfonylation of SarcoEndoplasmic Calcium ATPase (SERCA) at cysteine 674 (C674) was measured using a rabbit polyclonal anti-SERCA antibody raised against a peptide containing the sulfonylated cysteine 674 residue (C674) [20], [21], as we have described, in twenty images selected from 4 sections from each animal [13], [22].

### 2.4 RNA isolation, sequencing and gene set analysis

RNA was isolated from frozen LV, sequenced and analyzed as previously described [17], [18]. RNA was analyzed from the hearts of seven non-carrier, control-diet male mice; two non-carrier, SOTA-treated male mice; five E180G, control-diet male mice; and seven E180G, SOTA-treated male mice. Differential expression was assessed using the Wald test implemented in the DESeq2 R package. Correction for multiple hypothesis testing was accomplished in DESeq2 using the Benjamini-Hochberg false discovery rate (FDR) after excluding genes with low overall expression using the “independent filtering” step (the default behavior in DESeq2). All analyses were performed using the R environment for statistical computing (version 4.1.2). Gene Set Enrichment Analysis (GSEA; version 2.2.1) [23] was used to identify biological terms, pathways and processes that are coordinately up- or down-regulated with respect to experimental variables, as we have described [17], [18].

### 2.5 Data availability

The data discussed have been deposited in NCBI's GEO [24] and are accessible through GEO Series ID GSE315790.

### 2.6 Statistical analysis

All data are presented as mean ± SEM. Significance was determined using a 2-way repeated measures ANOVA and the Tukey’ test for multiple comparisons, except for transcriptomics, which is described above. GraphPad Prism (version 11.0.2) software was used for statistical analysis. A *p* < 0.05 was considered significant.

## 3 Results

### 3.1 Heart weights

Hearts were weighed at the end of 8 weeks of treatment. Heart weight normalized to tibial length was increased 16.5% in E180G mice, and the increase was prevented by SOTA treatment (Suppl. Fig. 1). The weight of the combined left and right atria was increased by 56.5% in E180G mice, and the increase was diminished by SOTA (Suppl. Fig. 1).

### 3.2 Echocardiography, left ventricular dimensions

At baseline total LV wall thickness was increased by 10.2% in E180G vs. NC mice and increased progressively at 4 and 8 weeks. SOTA treatment prevented the increase in wall thickness (Fig. 1**A** and Suppl. Fig. 2). The effects of SOTA on wall thickness were similar in male and female mice (Suppl. Fig. 2). LV end diastolic dimension, end systolic dimension, and fractional shortening were not different in E180G vs. NC mice at baseline, 4 or 8 weeks of treatment, and were not affected by SOTA treatment (Suppl. Figs. 3–5).

**Fig. 1:**
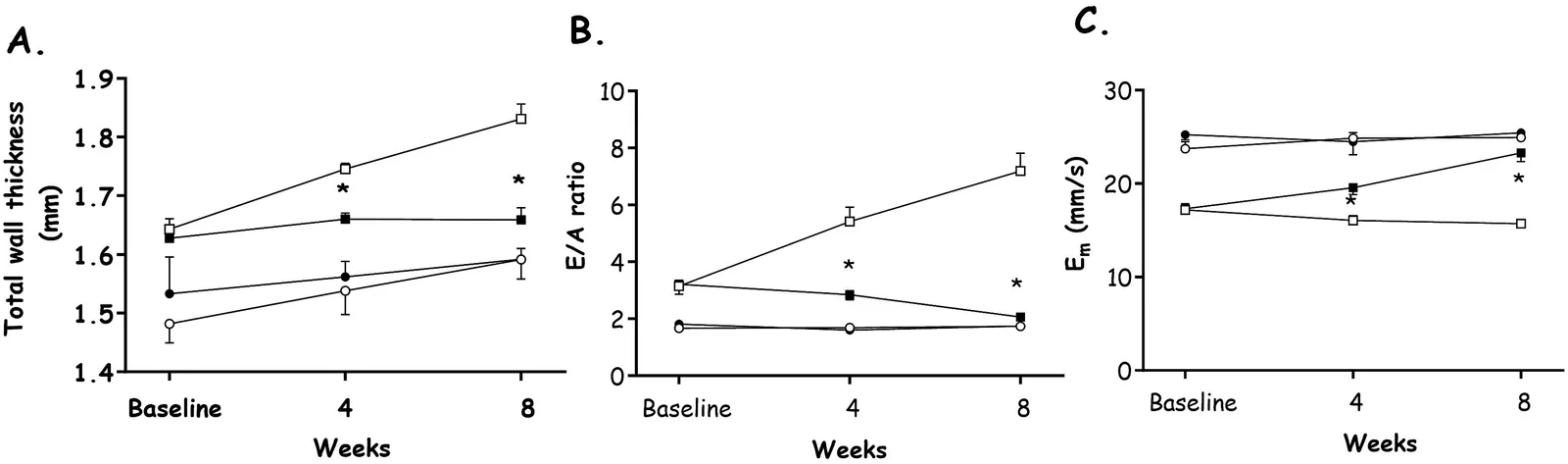
Effect of SOTA on LV hypertrophy and diastolic function in E180G mice. Male and female mice 7–8 weeks of age were randomized to control diet or diet supplemented with sotagliflozin (SOTA; 30 mg/kg) for 8 weeks. Echocardiographic measurements were made at baseline and after 4 and 8 weeks of treatment. Over the ensuing 8 weeks, LV wall thickness (**Panel A**) progressively increased in E180G mice, in association with a decline in diastolic function as reflected by an increase in E/A ratio (**Panel B**) and a decrease in Em (**Panel C)**. SOTA treatment prevented the increase in LV wall thickness and normalized both E/A and Em. ○, non-carrier, control diet (*n* = 6); ●, non-carrier (NC), SOTA (*n* = 3); ☐, E180G, control diet (CD; *n* = 8); ◼, E180G, SOTA (*n* = 11). Data shown are mean ± SEM for the indicated number of mice based on the average of at least 5 consecutive cardiac cycles per animal. *P < 0.05 vs. E180G CD by 2-way ANOVA. Echocardiographic data for individual animals at 8 weeks, including by sex, are shown in Suppl. Figs. 2–7.

### 3.3 Echocardiography, diastolic function

At baseline the transmitral flow velocity Doppler-measured E/A ratio was increased 47.6% in E180G vs. NC mice and increased progressively at 4 and 8 weeks. SOTA treatment prevented the increase in E/A, which was normalized by 8 weeks of treatment (Fig. 1**B**, Suppl. Fig. 6). Likewise, at baseline tissue Doppler-measured myocardial relaxation rate Em was depressed in E180G vs. NC mice, and the decrease was prevented by SOTA, which normalized Em by 8 weeks of treatment (Fig. 1**C** and Suppl. Fig. 7). The effects of SOTA on E/A and Em were similar in male and female mice (Suppl. Figs. 6 and 7).

### 3.4 Cardiac myocyte hypertrophy and interstitial fibrosis

At 8 weeks myocyte cross-sectional area was increased 36.8% in E180G vs. NC mice, and the increase was prevented by SOTA (Fig. 2). Myocardial interstitial fibrosis as assessed by Picrosirius red staining was markedly increased in E180G vs. NC mice, and the increase was mitigated by SOTA (Fig. 3).

**Fig. 2:**
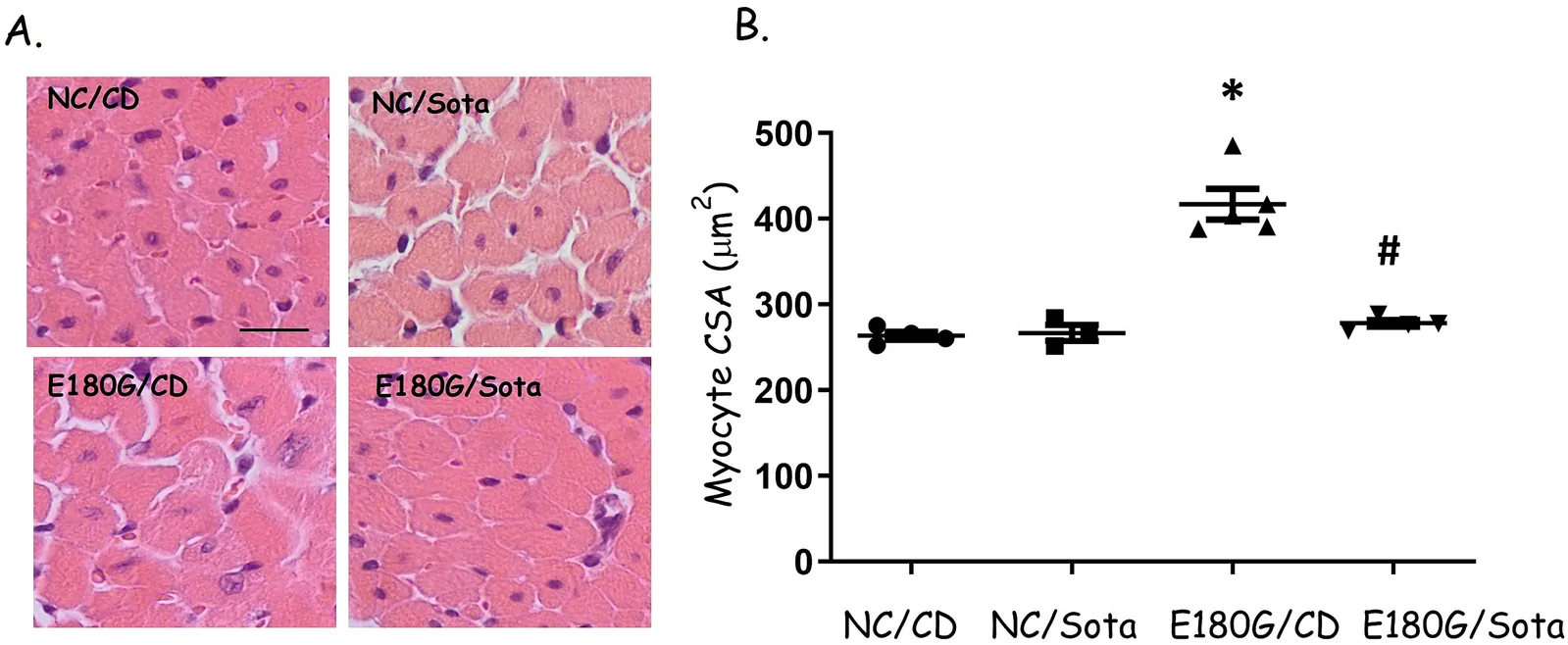
SOTA prevents myocyte hypertrophy. Male mice were sacrificed after 8 weeks of treatment and myocyte hypertrophy was assessed by measuring cross-sectional area (CSA). **Panel A** shows representative images (bar = 20 μm). Myocyte cross-sectional area was markedly increased in E180G mice, and the increase was prevented by SOTA. **Panel B** shows individual mouse data for mean myocyte cross-sectional area (± SEM). 60 myocytes were measured from 5 fields in each of 4 sections in each animal (*n* = 4 animals for NC/CD, 3 for NC/Sota, 5 for E180G/CD, and 4 for E180G/Sota). *P < 0.05 vs. NC CD or NC SOTA; ^#^*P* < 0.05 vs. E180G CD by 2-way ANOVA.

**Fig. 3:**
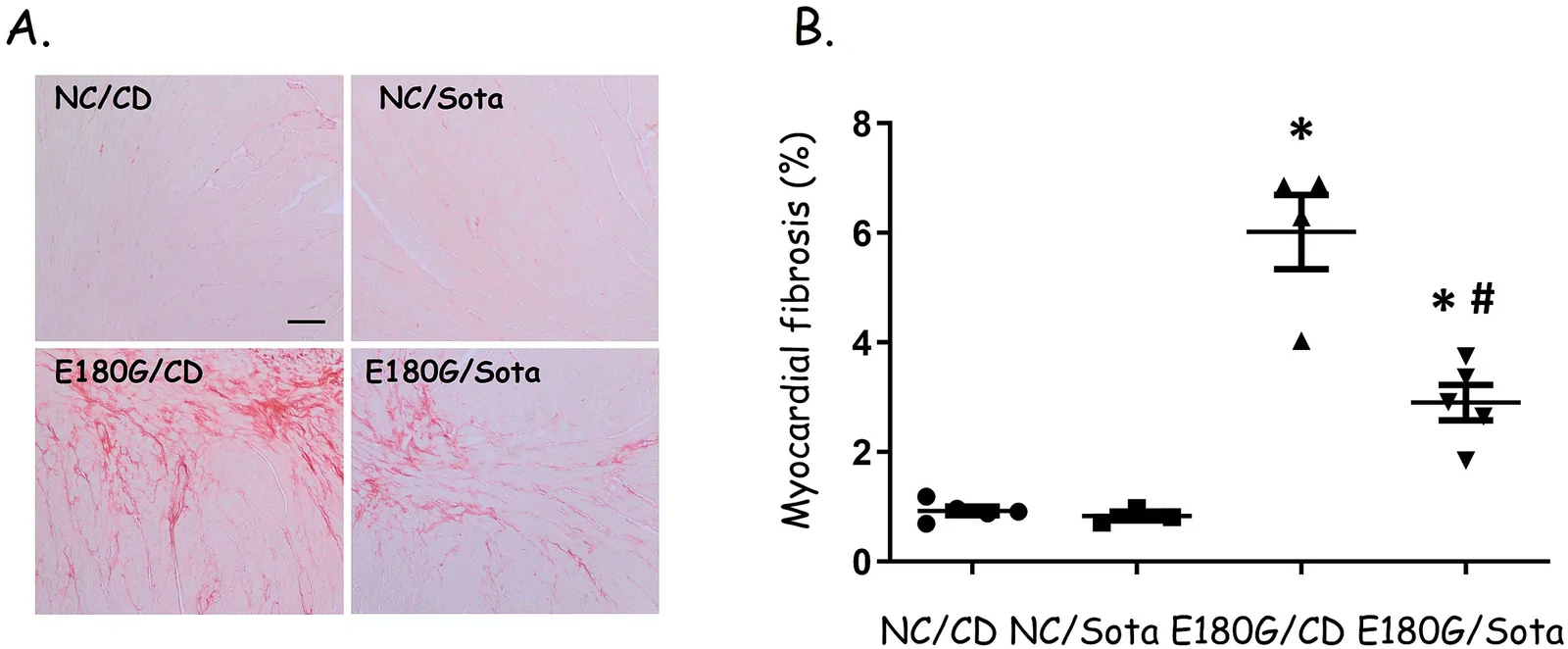
SOTA inhibits myocardial fibrosis. Male mice were sacrificed after 8 weeks of treatment and interstitial fibrosis was assess using picrosirius red staining. **Panel A** shows representative images (bar = 100 μm). E180G mice had marked interstitial fibrosis which was partially inhibited by SOTA. **Panel B** shows individual mouse data for mean percentage of interstitial fibrosis as a percentage of LV stained section area (±SEM). 4 random fields were quantified in each of 4 sections in each animal (*n* = 5 animals for NC/CD, 3 for NC/Sota, 4 for E180G/CD, and 5 for E180G/Sota). *P < 0.05 vs. NC CD or NC SOTA; ^#^*P* < 0.05 vs. E180G CD by 2-way ANOVA.

### 3.5 Myocardial oxidative stress and SERCA oxidation

Myocardial oxidative stress, as assessed by HNE, was markedly increased in E180G vs. NC mice and the increase was prevented by SOTA (Fig. 4). SERCA oxidative sulfonylation at C674 was markedly increased in E180G vs. NC mice, and the increase was abolished at 8 weeks of treatment (Fig. 5).

**Fig. 4:**
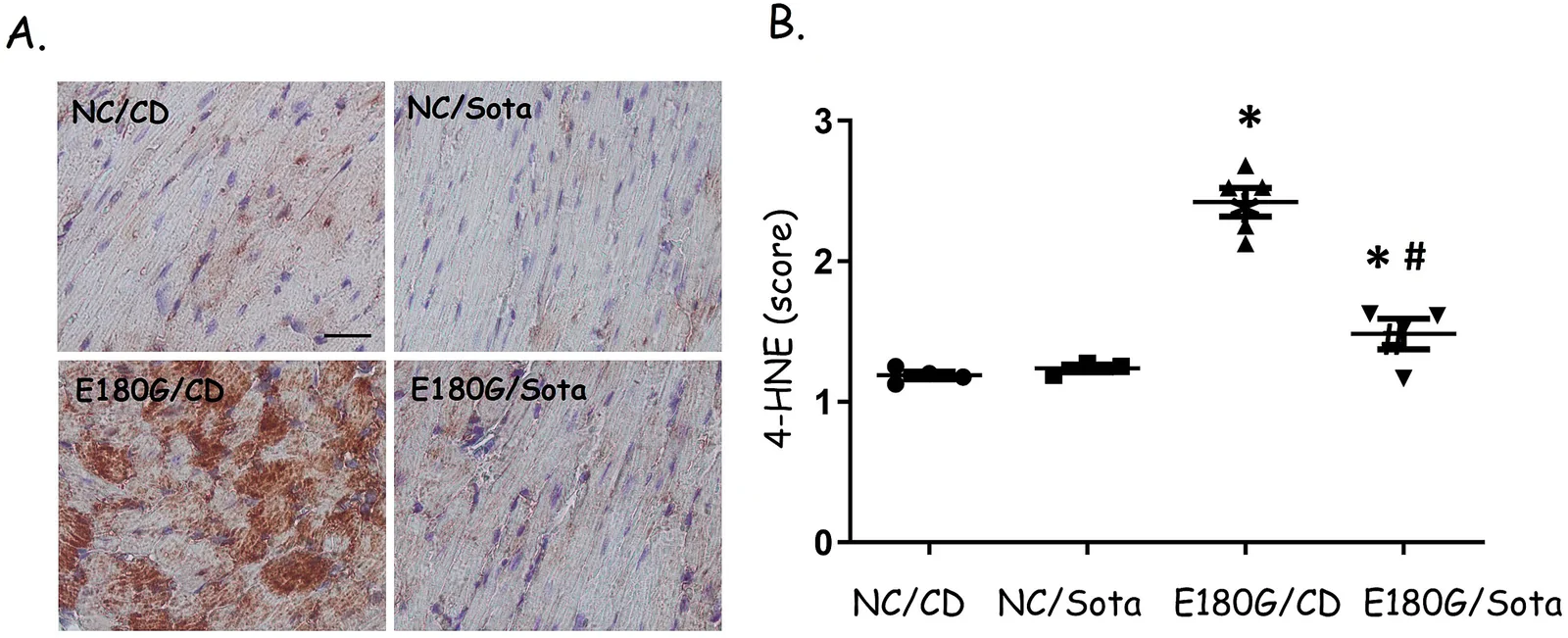
SOTA decreases oxidative stress in E180G mice. Myocardial oxidative stress was assessed histologically after 8 weeks of treatment using 4-hydroxynonenal (4-HNE) staining. **Panel A** shows representative images (bar = 25 μm). Oxidative stress was markedly increased in E180G mice, and was normalized by SOTA. **Panel B** shows individual mouse data for mean scoring for 4-HNE Staining (± SEM). Five random fields were selected in each of 4 sections in each animal (*n* = 4 animals for NC/CD, 3 for NC/Sota, 5 for E180G/CD, and 4 for E180G/Sota). *P < 0.05 vs. NC CD or NC SOTA; ^#^*P* < 0.05 vs. E180G CD by 2-way ANOVA.

**Fig. 5:**
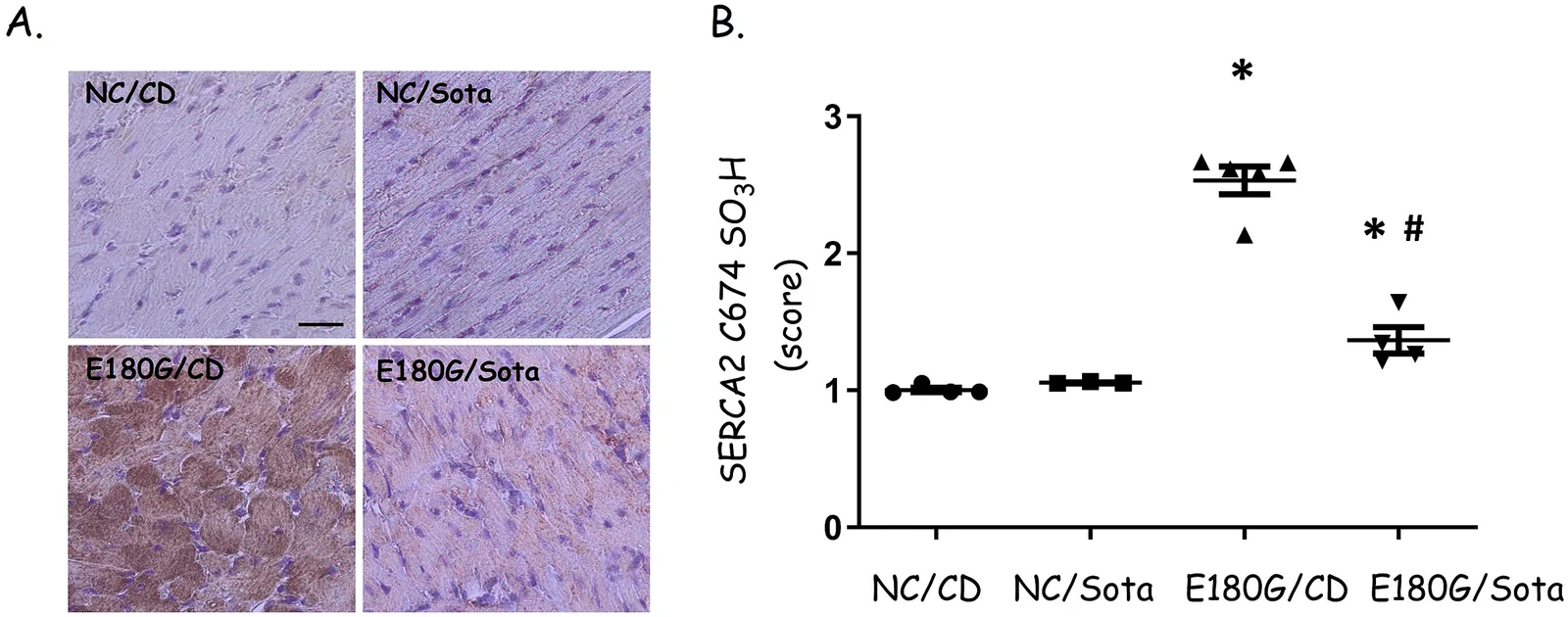
SOTA prevents oxidation of SERCA. Oxidative modification of SERCA by sulfonylation at C674 is irreversible and decreases SERCA activity. After 8 weeks of treatment SERCA C674 sulfonylation was assessed immunohistochemically using a site-specific antibody. **Panel A** shows representative images (bar = 25 μm). SERCA C674 sulfonylation was markedly increased in E180G mice, and the increase was prevented by SOTA. **Panel B** shows the graph with individual mouse data for mean scoring for SERCA2 C674 SO_3_H Staining (± SEM). Five random fields were selected in each of 4 sections in each animal (*n* = 4 animals for NC/CD, 3 for NC/Sota, 5 for E180G/CD, and 4 for E180G/Sota). *P < 0.05 vs. NC CD or NC SOTA; ^#^*P* < 0.05 vs. E180G CD by 2-way ANOVA.

### 3.6 Gene set expression analysis

Gene Set Enrichment Analysis (GSEA) was used to determine which gene sets from the Gene Ontology Biological Process (GOBP), Cellular Component (GOCC) and Molecular Function (GOMF) collections of the Molecular Signatures Database (MSigDB, v2024.1.Mm) demonstrated significant coordinate up- or downregulation with respect to each model effect or pairwise comparison. This analysis identified 316 gene sets that were coordinately downregulated with respect to genotype and 504 gene sets that were upregulated (FDR *q* < 0.05) (Suppl. File 1).

The GOBP, GOCC, GOMF, Hallmark and Reactome Pathway gene sets with the strongest coordinate downregulation by Normalized Enrichment Score (NES) in E180G hearts were all related to mitochondrial structure and/or function including cellular respiration, oxidative phosphorylation, electron transport and fatty acid oxidation. Table 1 (top) shows the 10 gene sets with the most decreased NES in HCM vs. NC (WT) hearts (NES ≤ −3.11, FDRq < 0.001 for all). With SOTA treatment all of these gene sets were upregulated (NES ≥ 2.75, FDRq <0.001 for all except Reactome mitochondrial protein degradation, FDRq < 0.25).

**Table 1:**
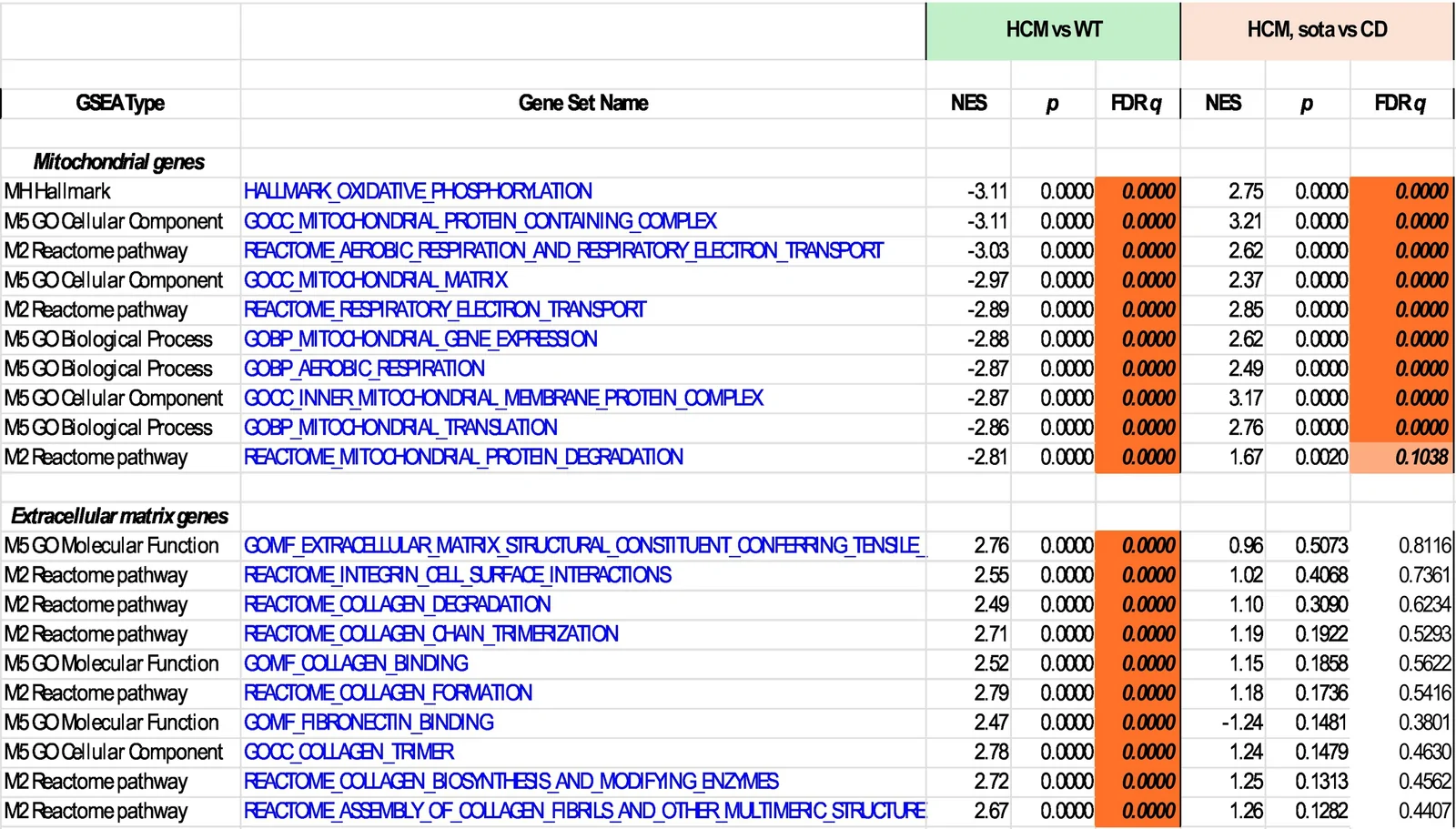
Gene sets for mitochondrial and extracellular matrix genes in E180G myocardium and the effect of SOTA treatment. Shown are the 10 most downregulated gene sets for mitochondria (top panel) and the 10 most upregulated gene sets (bottom panel) in HCM hearts as compared to wild type (WT) hearts based on normalized enrichment scores (NES) (*left column*). The *right column* shows the effect of SOTA vs. control diet in HCM mice. All ten mitochondrial gene sets were significantly upregulated by SOTA treatment for 8 weeks, whereas none of the extracellular matrix gene sets was significantly affected by SOTA treatment. RNA was analyzed from the hearts of 7 non-carrier, control-diet male mice; 2 non-carrier, SOTA-treated male mice; 5 E180G, control-diet male mice; and 7 E180G, SOTA-treated male mice. HCM, E180G; CD, control diet; P, nominal *p*-value; FDRq, false discovery rate;  = FDRq <0.001;  = FDRq <0.25).

The gene sets with the strongest coordinate upregulation in E180G hearts were related to fibrosis. Table 1 (bottom) shows the 10 GO gene sets with the most upregulated NES in HCM vs. NC (WT) hearts (NES >2.76, FDRq<0.001 for all). In contrast to the mitochondrial gene sets, SOTA had little or no consistent effect on the upregulated fibrosis pathways in HCM hearts (FDRq >0.25 in all).

## 4 Discussion

The present study had two goals. First, we tested whether the beneficial effects we observed with EMPA in the myosin R403Q mouse [1] can be replicated in a different mouse model and using a pharmacologically distinct SGLT2 inhibitor. Second, we tested whether the beneficial effects of SOTA are related to improvements in mitochondrial gene expression and oxidative stress. Our findings have implications for both the pathophysiology and therapy of HCM.

### 4.1 SOTA prevents or reverses the HCM phenotype in E180G mice

This study used the E180G mouse which is based on a known familial HCM mutation at the 180 codon leading to an amino acid substitution of glycine for glutamic acid of tropomyosin [2], a regulatory protein which modulates the interaction of troponin and myosin. The resulting modification causes a marked increase in myofibrillar calcium sensitivity, impaired diastolic function and myocardial remodeling with myocyte hypertrophy and interstitial fibrosis [3], [4], [5], [25]. In contrast, our prior study utilized the R403Q mouse model of HCM which is based on a mutation in the motor protein, myosin [1]. As expected, the E180G mice exhibited echocardiographic evidence of aggressive HCM with myocardial hypertrophy and diastolic dysfunction before treatment was started at 7–8 weeks of age [3], [4]. Over the subsequent 8 weeks of treatment, myocardial hypertrophy and diastolic dysfunction progressed, and the significance of the diastolic dysfunction was supported by an increase in atrial weight. Histologically, E180G mice had marked hypertrophy of cardiac myocytes and interstitial fibrosis. Treatment with SOTA arrested the progression of myocardial hypertrophy, decreased the amount of myocardial fibrosis, normalized diastolic function and decreased atrial size. While SOTA prevented the increase in wall thickness measured by echocardiography, the HCM/SOTA group remained numerically higher than the non-carrier group (p = ns). In contrast, SOTA normalized myocyte size measured histologically. This discrepancy may reflect the incomplete normalization of myocardial fibrosis at 8 weeks (Fig. 3). Myocardial hypertrophy and diastolic dysfunction were present in both male and female E180G mice. While the number of female mice precluded statistical analysis, the effects of SOTA were qualitatively similar in the two sexes.

These results extend our prior observation to a second HCM model. In contrast to our prior study which used EMPA, this study used the SGLT2 inhibitor SOTA. While EMPA and SOTA are both in clinical use for the treatment of cardiovascular diseases including heart failure, they differ markedly with regard to pharmacologic properties: EMPA is highly selective (2700-fold) for SGLT2 vs. SGLT1, whereas SOTA is a relatively non-selective (20-fold) inhibitor [6]. In addition, SOTA is significantly more lipophilic than EMPA [7]. These data thus extend our prior observations to a second, pharmacologically distinct SGLT2 inhibitor. Of note, SOTA is being evaluated in patients for the treatment of HCM [26].

### 4.2 SOTA upregulates mitochondrial gene expression

In E180G mice SOTA caused marked correction of down-regulated gene programs for both oxidative phosphorylation and fatty acid oxidation. Mitochondrial dysfunction plays a significant role in the pathophysiology of HCM [8], [9], [10]. Multiomic analyses of myocardium from patients with HCM have demonstrated downregulation of pathways for oxidative phosphorylation and fatty acid oxidation [10], [27], [28]. In the R403Q HCM mouse, we found that downregulation of gene and protein pathways for oxidative phosphorylation and fatty acid oxidation were associated with impaired energetics [1]. Likewise, in the E180G hearts the most downregulated gene sets encoded for mitochondrial structure and function, including oxidative phosphorylation and fatty acid oxidation. We previously saw similar upregulation of mitochondrial genes with ERTU and EMPA in other heart failure models [17], [18] and with EMPA in the R403Q HCM mouse [1]. In each of these models, upregulation of mitochondrial gene sets was associated with increased ATP generation.

### 4.3 SOTA decreases oxidative stress

A major consequence of mitochondrial dysfunction is increased release of ROS [16] which may adversely affect multiple cellular functions via oxidative modification of proteins, lipids and nucleic acids. HCM has been associated with oxidative modifications of proteins involved in the regulation of sarcomere function [15], calcium homeostasis [29] and mitochondria [16]. Mitochondrial dysfunction is a major source of pathologic ROS in the myocardium [30], [31], and ROS have been shown to cause myocyte hypertrophy and interstitial fibrosis in other models of heart failure [32], [33]. Likewise, in vitro ROS stimulate hypertrophy and fetal gene expression in cardiac myocytes [12] and collagen production in fibroblasts [34]. We therefore reasoned that the observed upregulation of mitochondrial gene expression by SOTA would decrease oxidative stress in E180G hearts.

To assess myocardial oxidative stress we measured HNE, a product of lipid oxidation in the myocardium, and oxidative modification of Sarco(endo)plasmic Reticulum Calcium ATPase (SERCA) using an antibody that is site-specific for sulfonylation of SERCA at C674 [20], [21]. In E180G hearts, HNE content and SERCA sulfonylation were markedly increased, indicative of oxidative stress, and both were normalized by SOTA. Oxidative modifications of functionally important proteins including SERCA, myosin-binding protein C [15], [16], [29], [35] and creatine kinase [16] have been observed in HCM patients and mouse models. SERCA S-sulfonylation at C674 causes irreversible inhibition of activity [13], [36], [37] and promotes degradation [21].

The ability of SOTA to decrease oxidative stress may reflect improved mitochondrial function leading to decreased generation of ROS. These findings also suggest that other strategies to decrease ROS may be effective in HCM [38]. For example, Solaro and colleagues showed that treatment of E180G mice with *N*-acetylcysteine (NAC) prevented the HCM phenotype and decreased oxidative modifications of myosin binding protein-C [38]. Likewise, using the overexpression of mitochondrial-directed catalase, Xu and colleagues were able to protect creatine kinase from deleterious oxidative modification in cardiac myocytes with an HCM genotype [16].

### 4.4 SERCA oxidation in HCM

Altered calcium regulation has been implicated in the pathophysiology of HCM based, in part, on the observation that SERCA protein levels and activity are decreased in myomectomy samples from patients [39], [40], [41]. Of note, in E180G neonatal mice gene transfer of SERCA [42] or crossbreeding with phospholamban knockout mice to increase SERCA activity [43] improves myocardial structure and function, suggesting that decreased SERCA activity contributes to the HCM phenotype. In mice with myocardial hypertrophy due to pressure overload we have shown that overexpression of catalase corrects SERCA activity, and ameliorates hypertrophy, fibrosis and diastolic dysfunction [44]. Taken together, these observations suggest that SERCA oxidation may contribute to impaired diastolic calcium handling in HCM, and further, that the beneficial effect of SOTA in HCM may be due, in part, to improved diastolic calcium handling.

### 4.5 SOTA decreases fibrosis

SOTA caused a marked decrease in the amount of myocardial fibrosis. In E180G hearts fibrosis was associated with marked upregulation of gene programs for fibrosis. However, unlike mitochondrial gene expression, which was partially corrected by SOTA, treatment had little or no consistent effect on the expression of fibrosis gene sets. This result indicates that the decrease in fibrosis with SOTA was mediated primarily at the post-transcriptional level. Since oxidative stress is a well-established stimulus for fibrosis [34], [45], [46], these findings raise the possibility that the decrease in fibrosis with SOTA was a consequence of the decrease in oxidative stress. Further studies will be needed to clarify this important point. The prevention of fibrosis was incomplete, possibly reflecting the limited 8-week treatment period relative to a more prolonged time course needed for resolution of fibrosis.

Our results are consistent with the demonstration in cTnT-Q92 HCM mice that treatment with NAC, which decreased myocardial oxidative stress as reflected by HNE and malondialdehyde, prevented myocardial fibrosis [47]. A small clinical trial of NAC in patients with HCM, however, did not detect significant effects on cardiac structure or function [48]. It is noteworthy that an increase in SERCA activity due to phospholamban knockout decreased myocardial fibrosis in E180G mice [43], suggesting that dysregulated calcium, a down-stream consequence of oxidative stress, may play a role in mediating fibrosis in HCM.

### 4.6 Limitations

There are limitations that should be recognized. First, although we demonstrated downregulation of mitochondrial gene programs, no direct measurements of mitochondrial function or ROS production were made, thus limiting our ability to conclude that the increased oxidative modifications we observed were mediated by mitochondrial ROS. Second, gene expression data might be affected by relative changes in cell types within the heart. However, gene sets relative to sarcomeric proteins (actin, actomyosin) were not affected by SOTA (data not shown), suggesting that the observed upregulation of mitochondrial genes was not due to a generalized increase in myocyte RNA. Third, due to the limited number of samples in the NC plus SOTA group (*n* = 2), analysis of the effect of SOTA in NC or the interaction of SOTA with genotype is not reliable and was not undertaken. Finally, the number of female mice was insufficient for adequately powered sex-specific analyses.

## 5 Conclusions

In E180G hearts SOTA decreased myocardial hypertrophy and fibrosis, normalized diastolic function, upregulated mitochondrial gene sets, and decreased oxidative stress and SERCA oxidation. These findings have two major implications. First, they suggest that SOTA may be of clinical value in the treatment of patients with HCM. It remains to be seen whether these effects of SOTA will provide clinical benefits that are complementary to myosin inhibitors. Second, our data show that the benefit of SOTA in HCM is associated with a decrease in oxidative stress. The mechanism by which SOTA led to a decrease in oxidative stress, and the relationship of the decrease in oxidative stress to the observed improvements in cardiac structure and function, remain to be determined. Taken together with prior observations of mitochondrial dysfunction in HCM [1], [10], our findings support the thesis that SOTA and drugs that improve mitochondrial function may be of value in the treatment of HCM.

## CRediT authorship contribution statement

**Fuzhong Qin:** Writing – review & editing, Methodology, Investigation, Formal analysis. **David R. Pimentel:** Writing – review & editing, Methodology, Investigation, Formal analysis, Conceptualization. **Tomas Baka:** Writing – review & editing, Methodology, Investigation. **Dominique Croteau:** Writing – review & editing, Project administration, Methodology, Investigation. **Richard A. Cohen:** Writing – review & editing, Supervision, Resources, Methodology, Conceptualization. **Ivan Luptak:** Writing – review & editing, Supervision, Resources, Methodology, Conceptualization. **Wilson S. Colucci:** Writing – review & editing, Writing – original draft, Validation, Supervision, Resources, Project administration, Methodology, Investigation, Funding acquisition, Formal analysis, Conceptualization.

## Disclosures

Supported in part by 10.13039/100000002NIH grants HL-064750 (WSC) and HL-166606 (IL); a Fellow-to-Faculty Award from the American Heart Association 15FTF25890062 (IL); and an investigator-initiated research grant from Lexicon Pharmaceuticals.

## Declaration of competing interest

The authors declare the following financial interests/personal relationships which may be considered as potential competing interests: Wilson S. Colucci reports financial support was provided by National Heart Lung and Blood Institute. Ivan Luptak reports financial support was provided by National Heart Lung and Blood Institute. Ivan Luptak reports financial support was provided by 10.13039/100000968American Heart Association, Inc. Wilson S. Colucci reports financial support was provided by Lexicon Pharmaceuticals, Inc. If there are other authors, they declare that they have no known competing financial interests or personal relationships that could have appeared to influence the work reported in this paper.

## Appendix A Supplementary data

Suppl. File 1 Gene set analysis.
