# Supplementary material for: Sotagliflozin corrects myocardial phenotype, mitochondrial gene expression and oxidative stress in mice with hypertrophic cardiomyopathy
Source: J Mol Cell Cardiol Plus. 2026 Oct 12;18:100873. doi: 10.1016/j.jmccpl.2026.100873 (PMC13694939; doi:10.1016/j.jmccpl.2026.100873)

Suppl. Figure 1. Individual animal data for heart and atrial weights at 8 weeks. Panel A. Heart weight/tibial length (HW/TL). **Panel B.** Combined left (LAW) and right (RAW) atrial weight normalized to tibial length. Values are mean ± SEM for the depicted number of animals (n = 6 for NC/CD, 3 for NC/Sota, 10 for E180G/CD, and 11 for E180G/Sota). *P<0.05 vs NC/CD; #P<0.05 vs. E180G/CD by 2-way ANOVA.

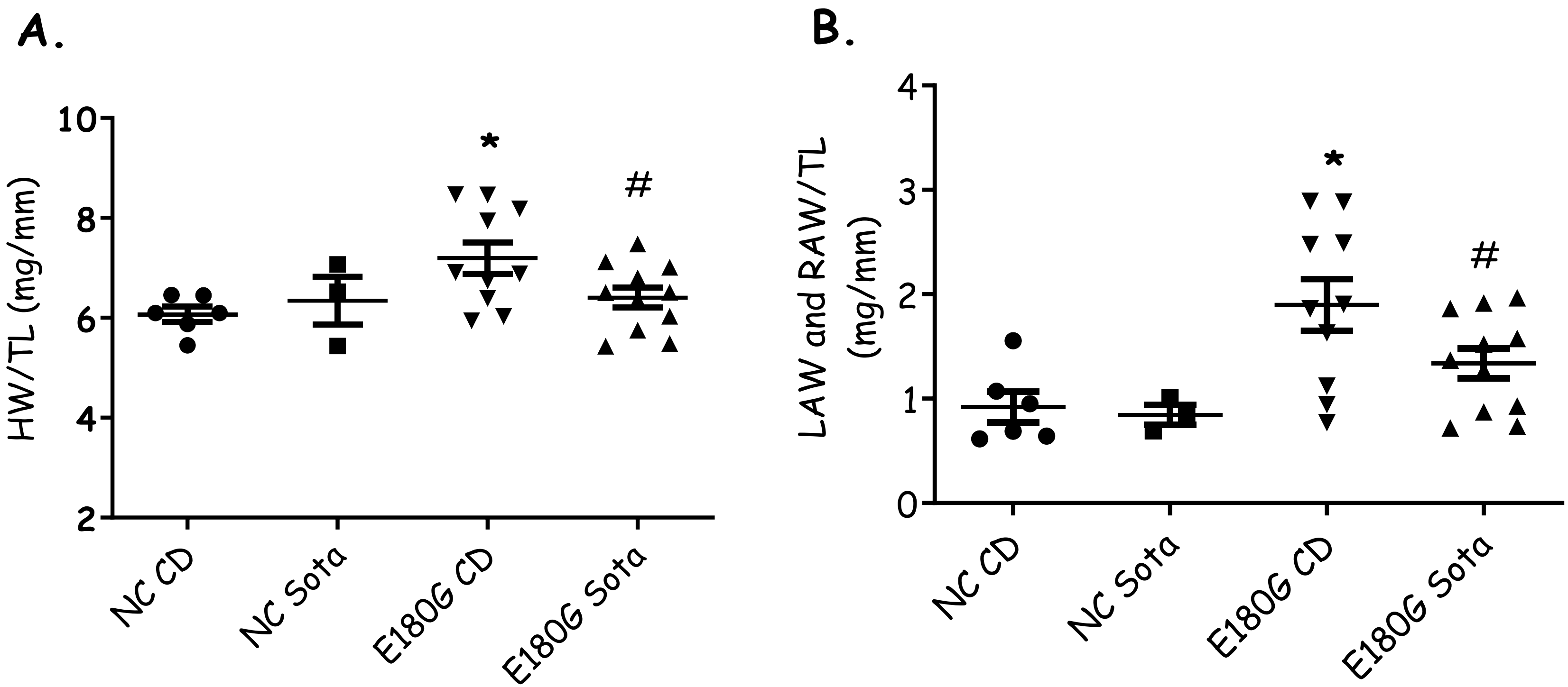

Suppl. Figure 2. Left ventricular total wall thickness. Individual animal data at 8 weeks for echocardiographic measurements of LV total wall thickness in the entire study population (**Panel A),** and the male **(Panel B)** or female **(Panel C)** cohorts. Values are mean ± SEM for the depicted number of animals. In Panel A, n = 6 animals for NC/CD, 3 for NC/Sota, 10 for E180G/CD, and 11 for E180G/Sota; in Panel B, n = 5 animals for NC/CD, 2 for NC/Sota, 7 for E180G/CD, and 7 for E180G/Sota; in Panel C; n = 1 animal for NC/CD, 1 for NC/Sota, 3 for E180G/CD, and 4 for E180G/Sota. *P<0.05 vs NC CD-B; #<0.05 vs NC CD-8W; &P<0.05 vs E180G CD-8W by 2-way ANOVA. Echocardiographic data in Figures 2-4 is based on the average of at least 5 consecutive cardiac cycles per animal.

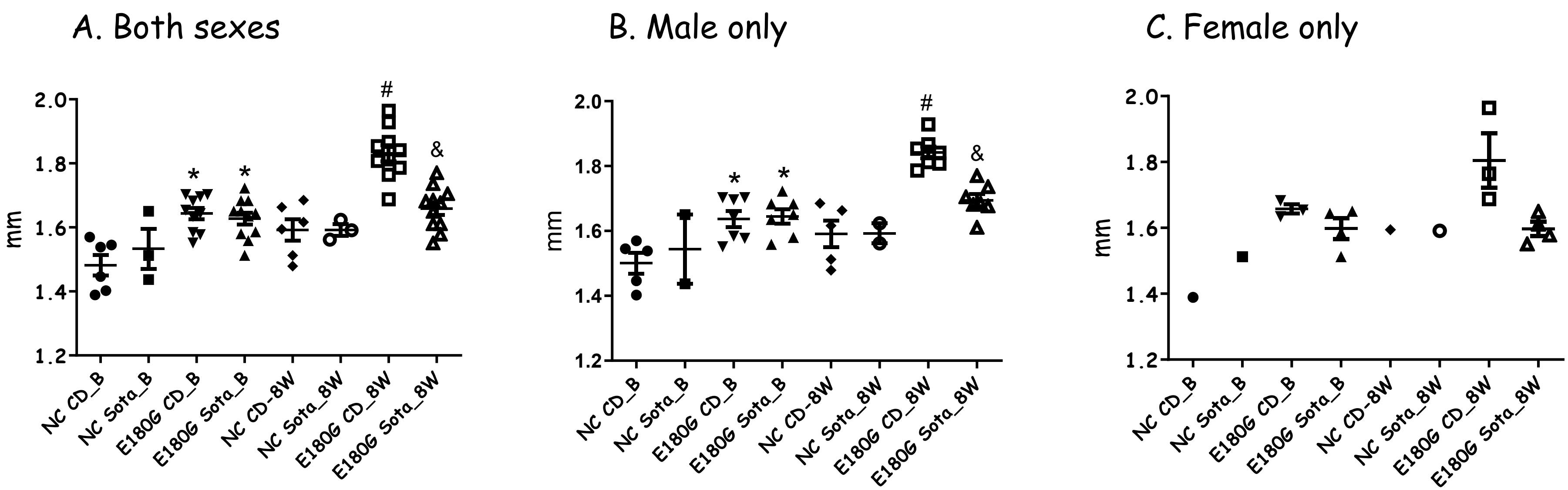

Suppl. Figure 3. Left ventricular end-diastolic dimension (EDD). Individual animal data at 8 weeks for the entire study population (**Panel A),** and the male **(Panel B)** or female **(Panel C)** cohorts. In Panel A, n = 6 animals for NC/CD, 3 for NC/Sota, 10 for E180G/CD, and 11 for E180G/Sota; in Panel B, n = 5 animals for NC/CD, 2 for NC/Sota, 7 for E180G/CD, and 7 for E180G/Sota; in Panel C; n = 1 animal for NC/CD, 1 for NC/Sota, 3 for E180G/CD, and 4 for E180G/Sota. Statistical analysis was by 2-way ANOVA.

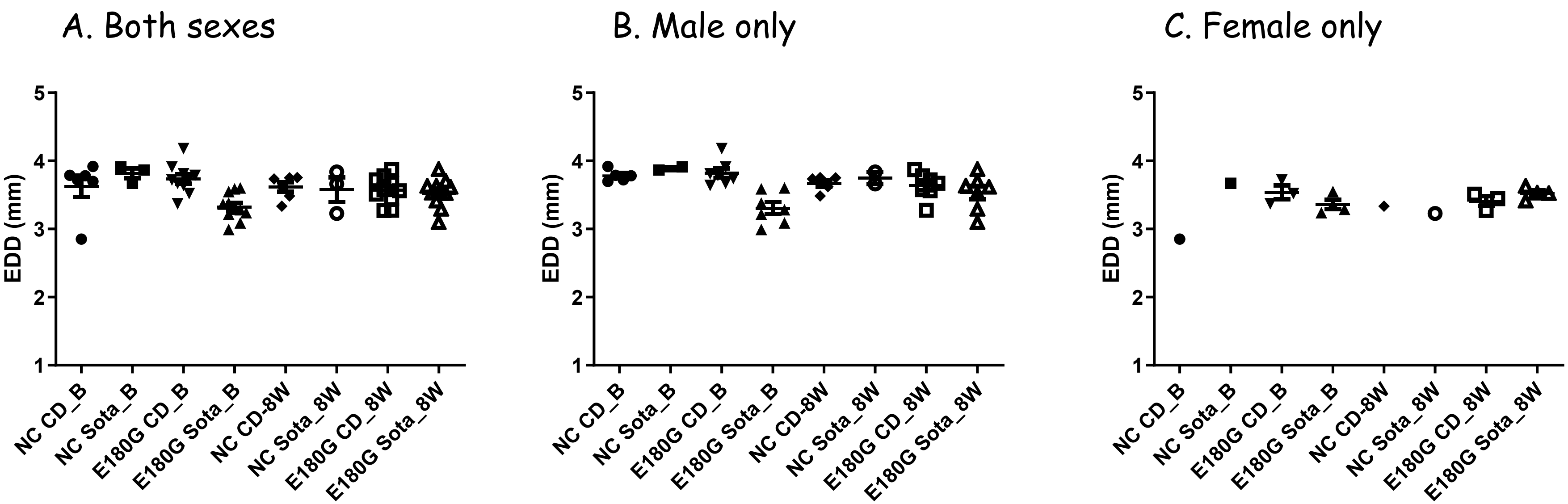

Suppl. Figure 4. Left ventricular end-systolic dimension (ESD). Individual animal data at 8 weeks in the entire study population (**Panel A),** and the male **(Panel B)** or female **(Panel C)** cohorts. In Panel A, n = 6 animals for NC/CD, 3 for NC/Sota, 10 for E180G/CD, and 11 for E180G/Sota; in Panel B, n = 5 animals for NC/CD, 2 for NC/Sota, 7 for E180G/CD, and 7 for E180G/Sota; in Panel C; n = 1 animal for NC/CD, 1 for NC/Sota, 3 for E180G/CD, and 4 for E180G/Sota. Statistical analysis was by 2-way ANOVA.

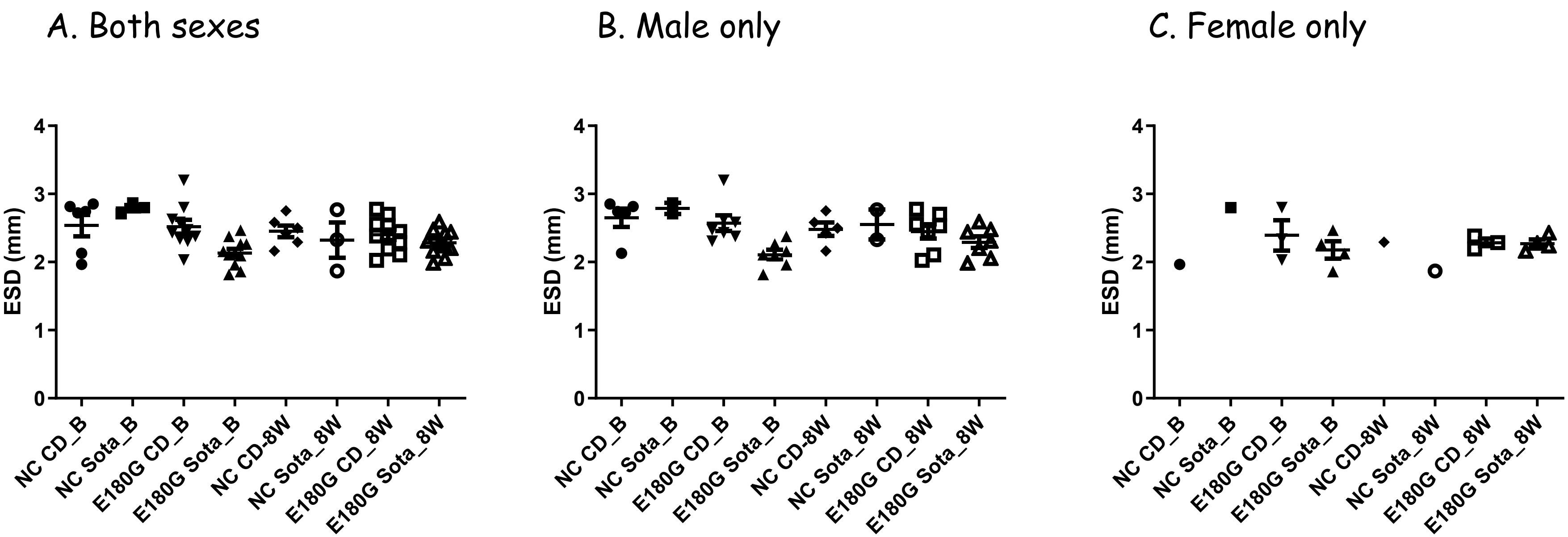

Suppl. Figure 5. Left ventricular fractional shortening (LVFS). Individual animal data at 8 weeks in the entire study population (**Panel A),** and the male **(Panel B)** or female **(Panel C)** cohorts. In Panel A, n = 6 animals for NC/CD, 3 for NC/Sota, 10 for E180G/CD, and 11 for E180G/Sota; in Panel B, n = 5 animals for NC/CD, 2 for NC/Sota, 7 for E180G/CD, and 7 for E180G/Sota; in Panel C; n = 1 animal for NC/CD, 1 for NC/Sota, 3 for E180G/CD, and 4 for E180G/Sota. Statistical analysis was by 2-way ANOVA.

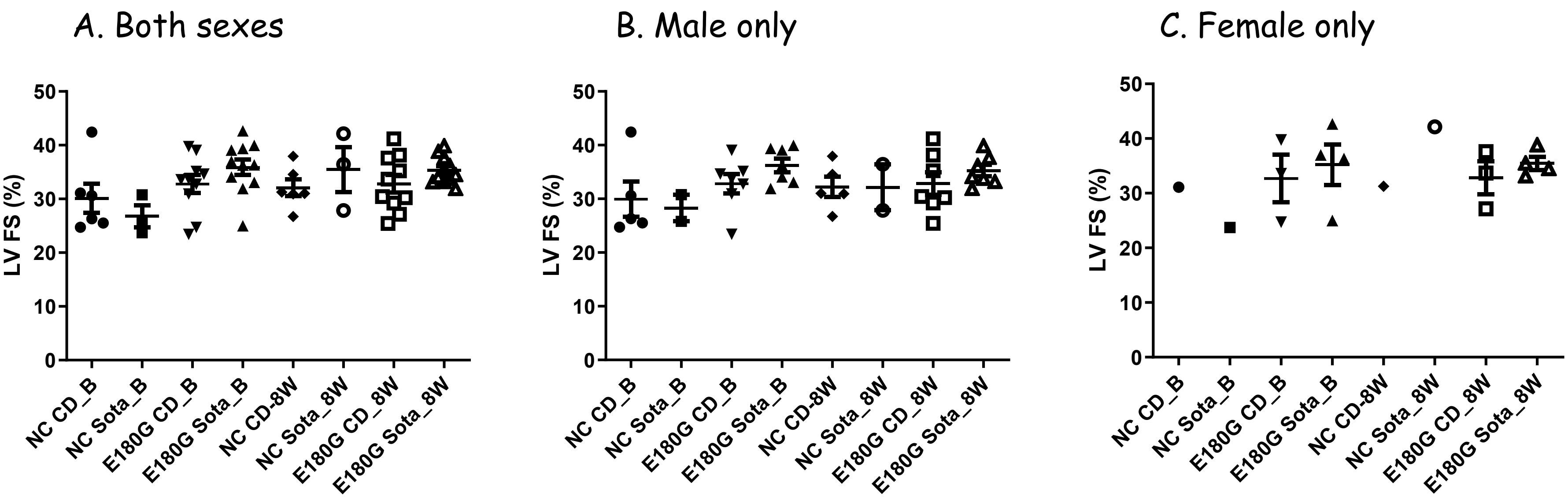

Suppl. Figure 6. Left ventricular E/A ratio. Individual animal data for echocardiographic measurements of E/A at 8 weeks in the entire study population (**Panel A),** and the male **(Panel B)** or female **(Panel C)** cohorts. Values are mean ± SEM for the depicted number of animals. In Panel A, n = 6 animals for NC/CD, 3 for NC/Sota, 10 for E180G/CD, and 11 for E180G/Sota; in Panel B, n = 5 animals for NC/CD, 2 for NC/Sota, 7 for E180G/CD, and 7 for E180G/Sota; in Panel C; n = 1 animal for NC/CD, 1 for NC/Sota, 3 for E180G/CD, and 4 for E180G/Sota. *P<0.05 vs NC CD-B; #<0.05 vs NC CD-8W; &P<0.05 vs E180G CD-8W by 2-way ANOVA.

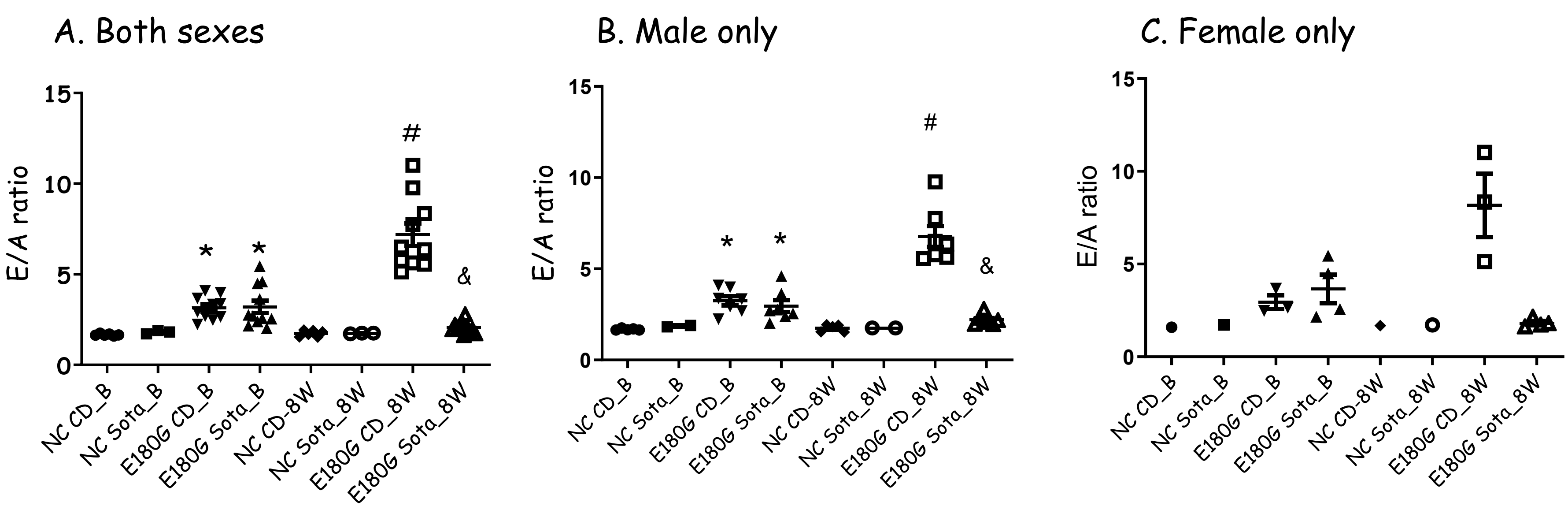

Suppl. Figure 7. Left ventricular Em. Individual animal data for echocardiographic measurements of Em at 8 weeks in the entire study population (**Panel A),** and the male **(Panel B)** or female **(Panel C)** cohorts. Values are mean ± SEM for the depicted number of animals. In Panel A, n = 6 animals for NC/CD, 3 for NC/Sota, 10 for E180G/CD, and 11 for E180G/Sota; in Panel B, n = 5 animals for NC/CD, 2 for NC/Sota, 7 for E180G/CD, and 7 for E180G/Sota; in Panel C; n = 1 animal for NC/CD, 1 for NC/Sota, 3 for E180G/CD, and 4 for E180G/Sota. *P<0.05 vs NC CD-B; #<0.05 vs NC CD-8W; &P<0.05 vs E180G CD-8W by 2-way ANOVA.

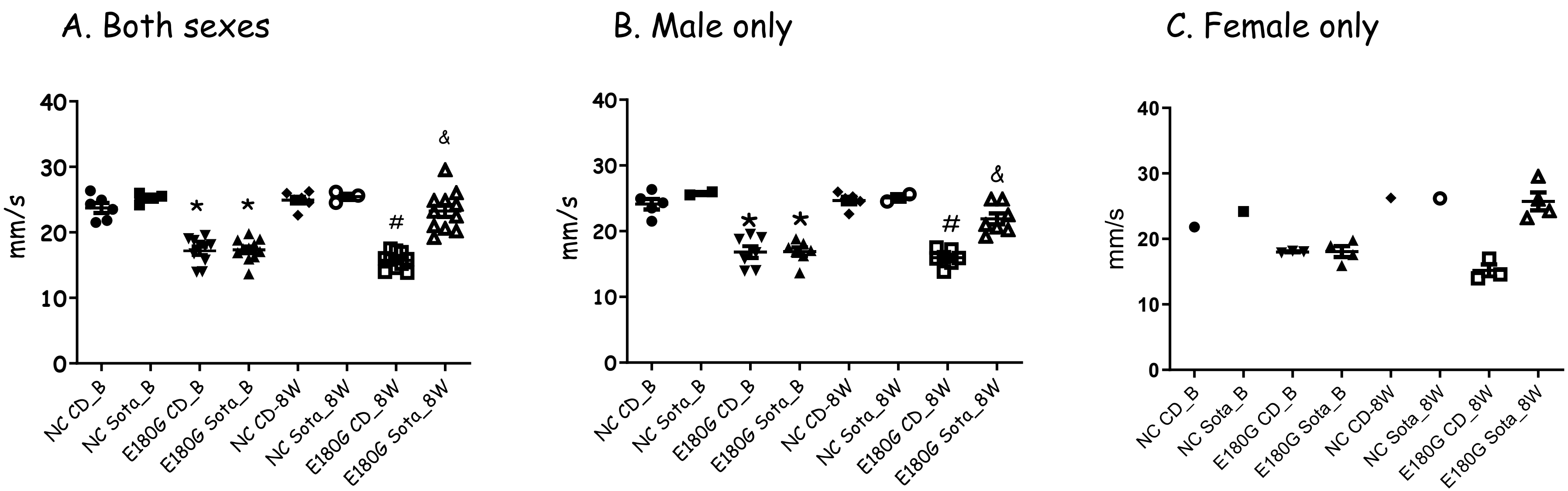

Supplement: Supplementary file 1 [file mmc2.pdf]
